# Immune checkpoint molecules in natural killer cells as potential targets for cancer immunotherapy

**DOI:** 10.1038/s41392-020-00348-8

**Published:** 2020-10-29

**Authors:** Yuqing Cao, Xiaoyu Wang, Tianqiang Jin, Yu Tian, Chaoliu Dai, Crystal Widarma, Rui Song, Feng Xu

**Affiliations:** 1 Department of General Surgery, Shengjing Hospital of China Medical University, 110004 Shenyang, China; 2 College of Life and Health Science, Northeastern University, 110819 Shenyang, China; 3 Lawrence D. Longo, MD Center for Perinatal Biology, Department of Basic Sciences, Loma Linda University School of Medicine, Loma Linda, CA 92350 USA

**Keywords:** Cancer, Immunology

## Abstract

Recent studies have demonstrated the potential of natural killer (NK) cells in immunotherapy to treat multiple types of cancer. NK cells are innate lymphoid cells that play essential roles in tumor surveillance and control that efficiently kill the tumor and do not require the major histocompatibility complex. The discovery of the NK’s potential as a promising therapeutic target for cancer is a relief to oncologists as they face the challenge of increased chemo-resistant cancers. NK cells show great potential against solid and hematologic tumors and have progressively shown promise as a therapeutic target for cancer immunotherapy. The effector role of these cells is reliant on the balance of inhibitory and activating signals. Understanding the role of various immune checkpoint molecules in the exhaustion and impairment of NK cells when their inhibitory receptors are excessively expressed is particularly important in cancer immunotherapy studies and clinical implementation. Emerging immune checkpoint receptors and molecules have been found to mediate NK cell dysfunction in the tumor microenvironment; this has brought up the need to explore further additional NK cell-related immune checkpoints that may be exploited to enhance the immune response to refractory cancers. Accordingly, this review will focus on the recent findings concerning the roles of immune checkpoint molecules and receptors in the regulation of NK cell function, as well as their potential application in tumor immunotherapy.

## Introduction

Natural killer (NK) cells are cytotoxic lymphocytes in the family of innate lymphoid cells that play essential roles in the first line of defense against cancer and viral infections.^1–3^ Human NK cells make up to 15 percent of the circulating lymphocytes and are associated with the killing and destruction of microbially infected and malignantly autologous and allogenic cells.^4^

NK cells have been determined to demonstrate antitumor cell cytotoxicity in the absence of prior sensitization and the subsequent production of cytokines alongside chemokines that have an effect on regulating certain immune responses.^4^ Some types of cancer, such as invasive breast carcinoma, bladder urothelial carcinoma, renal clear cell carcinoma, colon adenocarcinoma, lung squamous cell carcinoma, lower-grade glioma, pancreatic adenocarcinoma, stomach adenocarcinoma, cutaneous melanoma, uterine corpus endometrial carcinoma, and thyroid carcinoma, are associated with a deficiency of the number of NK cells leading to poor clinical outcomes.^5^ Many cancer types have demonstrated an imbalance of immune-regulated signals in NK cells. Inhibitory and activating receptors are expressed on the surface of NK cells and contribute to the execution of various NK cell functions.^4^ NK cells express the non-HLA-class I-specific inhibitory receptors (such as programmed cell death protein 1 [PD-1],^6,7^ T cell immunoreceptor with Ig and immunoreceptor tyrosine-based inhibition motif [ITIM]^8^ domains [TIGIT],^9,10^ a cluster of differentiation 112 receptor [CD112R], CD96, interleukin-1 receptor 8 [IL-1R8], and T cell immunoglobulin and mucin-domain-containing molecule 3 [TIM-3]), as well as activating receptors (such as NK group 2 [NKG2] family of receptor D [NKG2D] and coreceptor [CD226]).^4,11–13^ NK cells also express the HLA-class I-specific inhibitory receptors (such as killer cell immunoglobin-like receptor (KIR), NKG2A, and lymphocyte activation gene-3 [LAG-3]).^14^ Although more researches have shown that the blockade of checkpoint inhibitory receptors may be crucial in effectively reversing T cell exhaustion and the restoration of the antitumor capacity of T cells,^15^ there has been growing interest in the immune effects these treatments may have on NK cells as well.

Immune checkpoint inhibitors (ICIs) represent some of the most efficient immunotherapeutic approaches currently used to treat cancer.^16^ ICIs are antibodies that serve the role of binding to inhibitory molecules on the tumor-infiltrating surfaces of lymphocytes and thereby allowing the reactivation of antitumor immune responses.^17^ One of the promising discoveries to enhance antitumor activity and survival is the dual targeting of chimeric antigen-based receptor-engineered NK cells of glioblastoma, which has been found to overcome the heterogeneity of specific expression of target antigens and enhances the antitumor activity and survival.^17,18^ Moreover, immune checkpoint receptors (IC) contribute either positively or negatively in the regulation of activating the host immune response, preventing unwanted reactions that may affect healthy tissues.^19^

The term IC is mainly used about the inhibitory ICs critical in controlling NK and cytotoxic CD8^+^ T cells because of its high cytotoxic potential.^19^ Therefore, specific ICs are described concerning their regulation of either NK cell or T cell activity.^19^ NK cell activation is mainly regulated by receptors from the natural cytotoxicity receptor, NKG2, and KIR families.^19–22^ For example, activating and inhibitory KIR receptors serve to control the development and function of NK cell immunity while adjusting to the tumor microenvironment (TME).^23,24^ The interactions between KIRs and the corresponding HLA class I ligands contribute to the mediation of NK cell self-tolerance or cytotoxicity against the transformed cells.^24,25^ The control of unwanted damage of healthy cells by the NK cells is regulated by HLA-class I-specific receptors, which contain a fail-safe mechanism. Recent studies show that NKG2A forms a crucial checkpoint for controlling T cell and NK cell activation in cancerous conditions.^24,26^ The checkpoint receptors mediate the delivery of multiple signals, the balance of which determines if NK cells kill their target cells, such as stressed cells and tumor cells, or remain inactive.^27–29^

Dysregulation of checkpoint receptor signaling contributes to NK cell dysfunction.^30^ Furthermore, the downregulation of activating NK cell receptors and the upregulation of inhibitory receptors have been reported in multiple tumors. For example, NK cells expressed decreased levels of CD226 in various types of cancers, including myeloma, breast cancer, and myeloid leukemia.^14,15,31^ Notably, the downregulation of activating NK cell receptors, sometimes by helix-loop-helix protein ID2, serves as an active suppression and can be restored in the TME, such as by the repression of GSK3.^14,32,33^ As highlighted earlier, ICs can further be described as being membrane molecules that are mainly located, although not exclusively, on T lymphocytes and NK cells that act after recognizing appropriate ligands on the antigen-presenting cells (APC) or the target cells. Therefore, ICs can play either a negative or positive role in the processes of lymphocyte activation. The detrimental regulation of NK cell function also involves the upregulation of inhibitory receptors. For example, PD-1 upregulation was recently found in NK cells from multiple myeloma, Kaposi sarcoma, and head and neck cancer patients.^16,34–36^ TIGIT expression on NK cells was further upregulated in tumor regions compared with peritumoral regions in colorectal tumors.^10,24,37–39^ CD96, CD112R, TIM-3, NKG2A, LAG-3, and IL-1R8 are other significant receptors that have been proved to be upregulated on NK cells in cancers.^26,40,41^

Aside from this, the expression of various ligands on cancer cells has been reported to target common signaling molecules. The targeted interaction modifies the function of NK cells by regulating its numerous inhibitory and activating receptors. For instance, transformed cells may lose or decrease their HLA-I expression during cancer progression due to the activation of inhibitory NK cell receptors bound to their surfaces.^26^ Increased activation of inhibitory receptors and decreased stimulation of activating receptors reduce the antitumor response in the NK cells, and facilitate the induction of tumor immune escape. Several solid tumor types create an immunosuppressive microenvironment that prevents T cell or NK cell-mediated lysis through inhibitory ligands, such as the PD-L1 expressed on the surfaces of the cancer cells.^6,42–45^ The modulation of these checkpoint molecules and the development of inhibitory receptor blockade provide a vital strategy to improve NK cell function, such as by Diacylglycerol Kinase zeta.^46–49^

NK cells have essential roles in tumor surveillance and control. Some studies have identified the presence of dysfunctional NK cells in some cancer patients.^4,14,16,50^ Due to the inability to preserve their primary role, the dysfunctional NK cells allow certain cancers to escape the immune surveillance.^17,51,52^ Thus, restoring the function of NK cells in cancer patients may be a promising approach for immunotherapy.^53,54^ In the following sections, a detailed analysis of NK cell immune checkpoint receptors and their ligands will be discussed along with their prospective influence in cancer immunotherapy.^55–57^

## NK cell phenotype and function

**Fig. 1:**
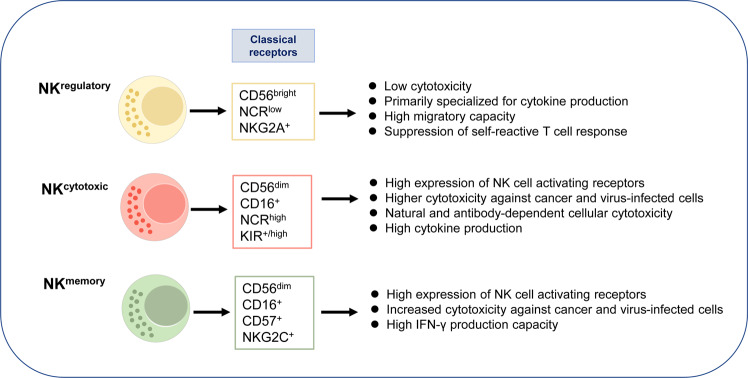
Phenotypical and functional properties of NK cells. According to classical receptors (for example, adhesion molecules-CD56 and CD57, activating receptors-CD16, NCR, KIR and NKG2C, inhibitory receptors-NKG2A), NK cells are subdivided into regulatory and cytotoxic phenotypes and memory NK cells. Therefore, these subsets of NK cells appear to be functionally different

NK cells display a range of phenotypic and functional characteristics (Fig. 1). The activated phenotype of NK cells is directly related to the progression of a disease. For example, there are phenotypic and functional differences that NK cells exhibit in patients with the immune reconstitution inflammatory syndrome compared with healthy individuals.^58^ The phenotype and cytotoxic function of NK cells are closely associated. Based on the experiment conducted to explore the extent to which NK cells in patients with cancers can be activated to kill cancer cells when stimulated by cytokines, NK cells have natural cytotoxicity that destroys cancer cells in cancer patients.^59^

On the other hand, cytokine-mediated cytotoxicity is regulated by a balance between the inhibitory and activating receptors. NK cells’ natural cytotoxicity requires co-engagement of several activating receptors such as KIR2DSs.^60^ Stimulated NK cells, such as tumor-primed cells, are phenotypically different from resting NK cells and characteristic with upregulation of NK cells’ expression of the inhibitory receptors and/or downregulation of the activating receptors.^59^

## Checkpoint receptors and ligands in NK cell dysfunction

**Fig. 2:**
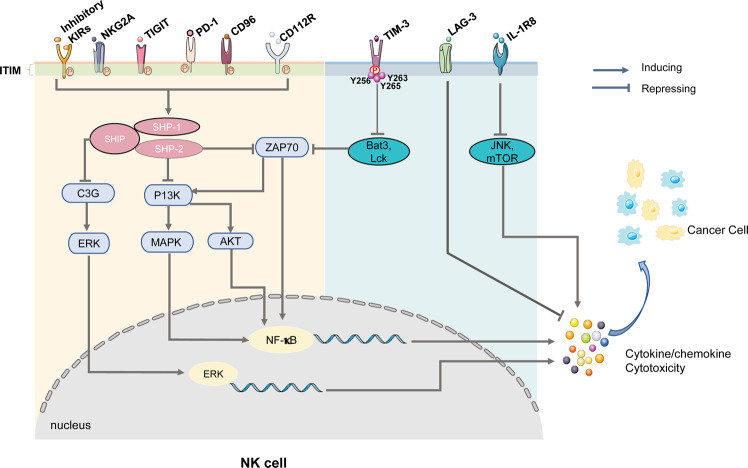
NK cell inhibitory receptor signaling is required for immune escape. Augmentation of inhibitory checkpoint NK cell receptor activation reduces the antitumor response in NK cells and facilitates the induction of tumor immune escape. In the tumor microenvironment, most of these receptors’ stimulation results in the phosphorylation of immunoreceptor tyrosine tail (ITT)-like motifs on their intracytoplasmic tails. After that, these immunoreceptor tyrosine-based inhibition motifs (ITIM) recruit phosphatases (such as Src homology domain-containing tyrosine phosphatase [SHP] and SH2 domain-containing inositol-5-phosphatase [SHIP]) that inhibit downstream signaling and ultimately suppress NK cell cytotoxic activity. Further research is necessary to understand better the signal mechanisms of these checkpoint receptors (including CD96, CD112R, and LAG-3). Nonetheless, studies have shown that these inhibitory signals attenuate NK cell cytotoxicity and antitumor cytokine/chemokine release, allowing tumor immune escape

**Fig. 3:**
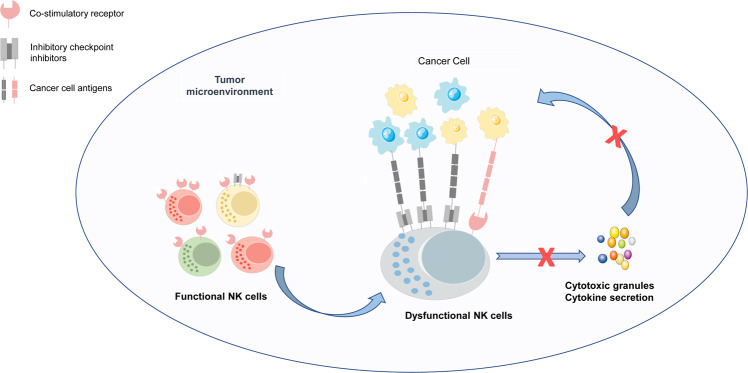
Schematic representation of a model of NK cell immune checkpoint receptors in tumor cell recognition. NK cell function is regulated and maintained by the balance of inhibitory and activating receptor signals. In the tumor microenvironment, NK cells transform to become dysfunctional cells with enhanced expression of inhibitory immune checkpoints and decreased expression of activating immune checkpoints. This imbalance of signaling transmission induces NK cell dysfunction and the extinction of antitumor immune response. Blockade of these inhibitory checkpoint molecules with the use of immune receptor inhibitors (ICI) (e.g., anti-KIR (lirilumab), anti-PD-1 (sintilimab and pembrolizumab), anti-NKG2A (monalizumab)), as well as activation of co-stimulatory receptor signaling by inducing co-stimulatory receptor expression (e.g., NKG2D ligand α3 domain-specific antibodies, cytokines (IL-2, IL-15, and IL-21), demethylating agents, histamine, anti-TGF-β monoclonal antibodies, TGF-β receptor inhibitors, TGF-β antisense oligonucleotides), can restore the antitumor activity of NK cells

As previously mentioned, NK cell function is regulated and maintained by the balance of inhibitory and activating receptors responsible for the preservation of the cell’s steady internal environment (cell homeostasis). In cancer microenvironments, the function of antitumor NK cells is affected by the decreasing expression of activating receptors and their ligands, as well as the increased expression of inhibitory immune checkpoint molecules; this facilitates the tumor immune escape (Figs. 2 and 3) and allows for the development of various cancer types.^61–64^ NK cell activation is primarily controlled by the dynamic balance between inhibitory and activating receptor signaling.^65^ As an example, NK cells express cell surface inhibitory receptors antagonizing to the activation pathways through protein tyrosine phosphatases.^66^ The nature of the typical inhibitory receptor, KIR, contributes to the recognition of self-antigens and subsequently provides negative signals that lead to the eventual suppression of NK cell activation through inhibition of the stimulatory signaling pathway. The ligand of the KIR is the major histocompatibility complex (MHC) class I molecule and is present in healthy cells. The inhibitory cell surface receptors are innately characterized by ITIM present in the cytoplasmic domains of various inhibitory receptors.^66^

### Killer cell immunoglobulin-like receptor (KIR) family

As highlighted earlier, NK cells across development express inhibitory KIR receptors that are specific for MHC class I antigens in a variegated and stochastic manner.^21,67^ KIRs are divided into haplotypes A and B, where A is more frequent and mainly constituted of inhibitory receptors, while B contains both types of receptors with a predominance of the activating type.^67^ The repertoire of NK cell KIR is highly dependent on both HLA and KIR polymorphisms.^21^ The inhibitory KIRs inhibit NK cell activity through ITIM-based recruitment of protein tyrosine phosphatases (SHP-1 and SHP-2) responsible for dephosphorylation of the surrounding tyrosine kinases and the adapter proteins, which include DAP-10.^67^ As a diversified and polymorphic group of NK cell receptors that comprise inhibitory and activating KIRs, each member of the KIR family recognizes a particular HLA class I allotype from -A, -B, -C as the ligand.^26^ As an example, the inhibitory KIR2DL1, KIR2DL2, and KIR2DL3 affiliate with HLA-C as their ligand, whereas HLA-A and HLA-B serve as the ligands attached to other KIRs that include the inhibitory KIR3DL2 and KIR3DL3.^68,69^ Furthermore, the KIRs, ligands for KIRs, and MHC-I molecules exhibit high natural polymorphism.^26^

### PD-1

In the context of cancer immunology, co-inhibitory signaling molecules are well described for T cells. One of the most notable is PD-1, also known as CD279. This receptor also occurs in NK cells and has been shown to reduce NK cell response.^70^ PD-1 is highly expressed on CD56^dim^ NK cells in the peripheral blood of approximately one-quarter of healthy humans.^71^ In ovarian carcinoma, the CD56^dim^ NK cells with high PD-1 expression have been found to have the phenotypic characteristics of fully mature NK cells with the NKG2A^-^KIR^+^CD57^+^ phenotype and are increased in patients.^72^ A recent study reported PD-1 expression by NK cells in the malignant pleural effusions of patients with primary mesothelioma or metastatic tumors, including lung cancer, intestinal adenocarcinoma, uterine cancer, breast cancer, and bladder carcinoma.^73,74^ Another recent study warned of the possibility of PD-1 leading promoting hyperprogression of cancer if amplified by PD-1 blockade,^36^ which can be the mechanism of how the receptor downregulates NK cell response.^75,76^

PD-1 binds to the ligands PD-L1 and PD-L2 to mediate inactivation of immune cells, including NK cells.^44,77,78^ Studies showed that PD-1/PD-L1 interaction in NK cells was required in MHC^+^ and MHC^–^ tumors. In mouse lymphoma tumor models, PD-1 inhibits NK cell-dependent immune surveillance and facilitates the escape of tumor cells from the NK cell response.^79–81^ More so, PD-1 contains an extracellular variable immunoglobulin (IgV) domain, a transmembrane region, and an intracellular tail with two phosphorylation sites; one phosphorylation site is in an immunoreceptor tyrosine-based inhibitory motif, and the other is in an immunoreceptor tyrosine-based switch motif.^72,82,83^ To be more specific, Y248 (a PD-1 tyrosine-based switch motif) interacts with SHP-2, which is a requirement of the inhibition of proximal signaling molecules (including ZAP70, PI3K/AKT, C3G, and ERK) that consequently leads to the suppression of immune cell activation.^79^ In contrast, the activity of NK cells can be enhanced by histidine-rich glycoprotein, which modulates PD-1 expression *via* anti-C-type lectin-like receptor 1B.^82,84^ These findings provide potential mechanisms involved in the upregulation of PD-1 in the peripheral blood NK cells of patients with Kaposi sarcoma, NK cells from ovarian cancer ascites, and in the peripheral and tumor-infiltrating NK cells of patients with digestive cancer.^76,85–89^

### TIGIT and CD96

Two additional inhibitory receptors, CD96 and T cell immunoreceptor with Ig and ITIM domains (TIGIT), bind to the DNAM-1 ligand and serve to oppose DNAM-1 function.^90^ TIGIT, also known as WUCAM and Vstm3, is an immune checkpoint molecule that inhibits the activation of T cells and NK cells.^7,91–95^ It contains an IgV domain, a transmembrane domain, and an immunoreceptor tyrosine-based inhibitory motif (ITIM).^92^ TIGIT has the capacity of disrupting DNAM-1 significantly through *cis* interactions to form heterodimers. Subsequent blockade of TIGIT with monoclonal antibodies augment the antitumor and antiviral activity of NK cells and T cells based on studies on mouse models.^96,97^ The expression of TIGIT plays a vital role in suppressing activation and maturation of NK cells.^92,98–100^ Therefore, TIGIT has a role in tumor immunosurveillance, similar to the role of the PD-1/PD-L1 axis during tumor immunosuppression.^44^

Studies have shown that the interaction of TIGIT with the poliovirus receptor (PVR) and poliovirus receptor-like 2 (PVRL2), also named CD112, Nectin-2, and PRR2, directly inhibits NK cell cytotoxicity.^92,101,102^ In addition, TIGIT has immunosuppressive effects, in that it competes with DNAM-1 for nectin-like ligands. An excellent example of the nectin-like ligand is CD155, the primary ligand for TIGIT. CD155 is expressed in many types of cancer cells.^103^ As highlighted, the intracellular domain of TIGIT consists of an immunoreceptor tyrosine tail (ITT) and ITIM.^10^ ITT–like motifs play a crucial role in inhibiting signals. The engagement of TIGIT with CD155 encourages its phosphorylation through the Src-family kinases Fyn and Lck; this results in the recruitment of SHIP-1, which in turn downregulates the PI3K, MAPK and NF-κB signaling pathways in modulating immune cell function.^92,104,105^ TIGIT can be readily detected on resting human NK cells but not on mouse NK cells. The engagement of TIGIT with CD155 prevents human NK cytotoxicity and cytokine production; this is made possible by counterbalancing DNAM-1 mediated activation, which can be reversed by antibody-mediated TIGIT blockade.^106,107^ The blockade of TIGIT makes NK cells resistant to inhibition by myeloid-derived suppressor cells.^96,108^ In like manner, a recent study showed that downregulated TIGIT expression inhibited the proliferation of colorectal cancer cells.^37,109,110^

CD96, also known as TACTILE (T cell activation, increased late expression), is a member of the immunoglobulin gene superfamily and an immune inhibitory receptor expressed on resting NK cells.^111–115^ The protein, CD96, facilitates adhesion of NK cells and T cells during immune responses.^114^ CD96 is similar to TIGIT, based on its competition with DNAM-1 for nectin and nectin-like ligands, and inhibits the activity of NK cells.^116,117^ The binding of CD96 to CD155 inhibits IFN-γ production by NK cells.^111,118^ Furthermore, studies of metastatic lung tumors in the mouse model demonstrated that the antibody-mediated blockade of CD96 promoted IFN-γ production by NK cells and improved the control of this cancer.^111,119,120^ The effect of antibody-mediated blockade of CD96 on NK cell function and its impact on human cancer patients remains unknown; thus, further study is needed to understand its potential as a targeted molecule for immunotherapy.

### TIM-3

TIM-3 is in the TIM family of receptor proteins, all of which have similar structures. The extracellular regions of TIMs consist of a single membrane-bound distal IgV domain and a glycosylated mucin domain of variable length that is close to the membrane.^12,121–123^ The intracellular domain consists of a C-terminal cytoplasmic tail with five conserved tyrosine residues that interact with multiple components of the T cell receptor complex.^124,125^ TIM-3 is expressed on nearly all mature CD56^dim^CD16^+^ NK cells and is upregulated following stimulation with IL-12 and IL-18, and by IL-15 mediated NK cell maturation;^126–128^ this suggests that TIM-3 acts as a marker for NK cell maturation or activation, and TIM-3 cross-linking can inhibit NK cell-mediated cytotoxicity.^129,130^ Importantly, upregulation of TIM-3 has been found in gastric cancer, lung cancer, renal cancer, head and neck cancer, melanoma, schwannoma, follicular B-cell non-Hodgkin lymphoma, cervical cancer, prostate cancer, colorectal cancer, urothelial bladder carcinoma, esophageal cancer, and in the peripheral blood of patients with ovarian cancer.^35,93,131–143^ The co-expression of PD-1 and TIM-3 correlates with acute myelogenous leukemia progression, suggesting that TIM-3 has potential as a prognostic biomarker for various cancers.

Some of the cognate ligands for TIM-3 include galectin-9, phosphatidylserine, high mobility group protein 1, and carcinoembryonic antigen-related cell adhesion molecule 1.^126,144–148^ TIM-3’s cytoplasmic tail lacks a classical signaling motif and is replaced by five conserved tyrosine residues where some of them serve for signaling, and they include Y256, Y263, and Y265.^148,149^ The tyrosine residues signal through regulated interaction with HLA-B associated transcript-3 (Bat3). Bat3 binds to TIM-3 while in a steady-state, and sequentially recruits Lck, which is catalytically activated and promotes T cell signaling.^150,151^ Y256 and Y263 become phosphorylated, leading to the dissociation of Bat3; this, in turn, promotes T cell inhibition.^150,151^ TIM-3 is conservatively increased on both resting and activated T cells. In contrast, TIM-3 is only marginally increased on NK cells to oppose NK cell activation.^152^ Therefore, further research should better determine the role of TIM-3 in tumor surveillance to better understand its potential application in NK cell-mediated cancer treatment.

TIM-3 targeting effects do not affect non-tumor bearing tissues, although it does not seem to have any specificity.^143^ This remains one of the cancer research mysteries that although specificity remains a crucial factor in regulating immune responses in tissue.^122,153,154^ The upregulation of TIM-3 in several types of cancer raises concerns about the safety of all other tissues. However, research has shown that the safety of NK cells may be improved by irradiation.^126,155^ TIM-3 ligands expressed in tumor tissues, such as the Ceacam-1 and galectin-9. The effect of the TIM-3 blockade on tumor tissue is preferential.^131,156^ Therefore, targeting TIM-3 in a person with neck cancer will only affect other tissues if those tissues have some known ligands of the TIM-3 blockade.^157^ TIM-3 blockade is regulated by functional specification, a concept through which pathways such as the TIM-3 pathway sometimes regulate immune responses’ distinct features; thus, some other tissues in a patient with the neck cancer can remain unaffected by the targeting.^134,156,157^

### CD112R

CD112R, also known as poliovirus receptor-related immunoglobulin domain protein (PVRIG), is a newly identified inhibitory checkpoint receptor in the PVR-like family. CD112R is constitutively expressed on mouse NK cells, natural killer T cells, and CD8^+^ T cells, and also expressed on human T cells and NK cells.^101,158–160^ Based on research findings, the expression of CD112R on CD16^+^ NK cells from human PBMCs was nearly equal to that on CD16^−^ NK cells.^160^ CD112R was also highly expressed on NK cells from ovarian, endometrial, kidney, prostate, lung, and breast cancers.^11^ The receptor interacts with PVRL2 but not PVR (CD155) and competes with CD226 for PVRL2 binding to inhibit T cell and NK cell function.^11,158,160^ Human tumors with high PVRL2 expression and lower PVR expression are more likely to respond to CD112R blockade ex vivo.^11^

In the CD112R-deficient mouse TME of colon carcinoma and melanoma, increased CD8^+^ T cell effector function inhibited tumor growth compared with wild-type mice. Furthermore, it has been established that anti-CD112R antagonists combined with anti-PD-L1 could also reduce tumor growth or metastasis.^155,158,161,162^ In an ex vivo study on human tumor-infiltrating lymphocytes, CD112R blockade enhanced T-cell function, and in combination with TIGIT or PD-1 blockade, further enhanced the effect.^11,163^ However, its function in human NK cells is still inadequately known. In a preclinical study, it was established that the blockade of CD112R alone or together with TIGIT-blockade enhances the trastuzumab-triggered antitumor response in human NK cells and improves the efficacy of trastuzumab therapy for breast cancer.^104,160,164^ This finding suggests that CD112R on NK cells may be a potential target for breast cancer treatment in the future, although its efficacy and safety remain to be further verified through animal experiments and clinical trials.

### IL-1R8

Interleukin-1 receptor 8 (IL-1R8) is also called a single immunoglobin IL-1-related receptor and is a member of the IL-1 receptor family. It has functional and structural characteristics that negatively regulate ILR and Toll-like receptor.^75,165,166^ IL-1R8 has been identified as a checkpoint protein in NK cells that regulate antitumor activity in solid cancers. The peripheral blood of Il1r8^−/−^ mice proved to have an increase in NK cell number and maturity. The attenuation of IL-18 destroyed NK cell maturation in Il1r8^−/−^ cells.^75,167^ The deficiency of IL-1R8 can also result in enhanced IL-18-dependent activation of the JNK and mTOR pathways essential for the control of NK cell differentiation, activation, and metabolism. NK cells with low IL-1R8 levels tend to sustain more extended activation after stimulation. These NK cells also indicate high antitumor immunity levels (like IFN-γ); whereas, small pro-inflammatory cytokine (CXCL1, IL-6, CCL2, IL-1β, and TNF-α) levels attribute to promoting tumor growth.^142,167,168^ Importantly, a genetic IL-1R8 blockade can result in NK cell-mediated resistance to hematogenous liver metastasis, hepatic carcinogenesis, and lung metastasis.^108,167^ Interventions to overcome this resistance are already in their various testing stages; chimeric antigen receptors (CARs) engineered NK cells are one of them that has been found to be sufficiently effective.^169–173^ These findings show that IL-1R8 is an NK cell checkpoint protein that may be inhibited to promote antitumor activity.

### LAG-3

Structurally, LAG-3 is similar to CD4, yet it manifests a higher binding affinity to MHC class II molecules than CD4. It is expressed on activated T cells and NK cells.^174^ Another of its potential ligand is LSECtin, a member of the DCSIGN family.^175,176^ There is a high expression of LAG-3 in patients with Hodgkin lymphoma, acute myelocytic leukemia, and chronic lymphocytic leukemia.^177–179^ Its cytoplasmic tail is made up of three unique and conserved regions in mice and humans. It includes a serene phosphorylation site, a glutamic-acid proline (EP) repeats, and a KIEELE motif. Of the three, the KIEELE motif is the one that serves as the inhibitory function of LAG-3 in CD4^+^ cells.^180^ The effector function of T cells is inhibited by the engagement of LAG-3 and enhanced by the blockade of LAG-3.^180,181^ It is interesting to learn that LAG-3 is involved in the exhaustion of T cells as a result of pathophysiological basics; hence its combination with PD-1 synergizes to reinstate T cell function.^182,183^ The role of LAG-3, however, in regulating NK cell function is not yet clear and therefore calls for more research and investigation. NK cells from LAG-3 deficient mice show defects in the killing of specific cancer cells.^184^ It is worth noting that blocking the LAG-3 pathway with the use of LAG-3 antibodies or even soluble LAG-3 does not have any effect on the cytotoxicity of human NK cells.^176,185^ Even so, targeting LAG-3 may be useful in immunotherapy due to its influence on T cell and NK cell effector function.

### NKG2A

NK group 2 member A (NKG2A) is a NK cell receptor of the NKG2 family, a type II membrane receptor that forms a heterodimer with CD94.^16,28^ It dimerizes with CD94 to form an inhibitory receptor that is related to C-type lectins and recognizes HLA-E (also known as MHC class I antigen E).^28^ These inhibitory receptors interact with MHC I ligands on target cells, leading to complete inhibition of NK cell granule polarization and the prevention of cytotoxic granule release.^186^ NKG2A contains two immunoreceptor tyrosine-based inhibition motifs (ITIMs) in its cytoplasmic tail. These ITIMs are phosphorylated following ligation of the ITIM-bearing receptor and facilitate this leads to the recruitment of tyrosine phosphatases, such as SH2 domain-containing phosphatase (SHP)-1 and SHP-2.^187^ The recruitment of SHP-1 by the ITIM-bearing receptors seems to inhibit the initiation of signaling in that it blocks most downstream signals in NK cells.^187^ The selective dephosphorylation of Vav1, the only protein detectably associated with the catalytic site of SHP-1, by SHP-1 leads to the inhibition of NK cell cytotoxicity, whereas the phosphorylation of an adapter protein CRK leads to the inhibition of NK cell function after ligation of NKG2A to HLA-E.^188–190^ Previous studies have shown that NK cells express NKG2A in cancers of the breast, cervix, liver, and lung.^191–194^ Importantly, tumor immunity increases when the inhibitory NKG2A receptor is blocked. The strong capability of NKG2A to suppress NK cells means that the blockade of NKG2A will effectively lead to the restoration of NK cells. For example, the blockade of NKG2A in an experiment using an anti-NKG2A antibody against NKG2A ligand in mice led to the effective alleviation of the functional impairment of NK cells.^98,195^ The prospective use of immune checkpoint inhibitors, as demonstrated by NKG2A, propels the development of cancer immunotherapy.^42,196–198^

## Activating immune checkpoint receptors

Some additional co-stimulatory pathways are involved in regulating NK cell responses, which impede the development of cancer and contribute to cancer immunotherapy. For example, CD226, also known as PTA-1 or DNAM-1, is an activating immune receptor that is expressed on NK cells, which has cytolytic activity and functions in lymphokine secretion.^116^ It contains two Ig-like domains on its extracellular portion and has a cytoplasmic tail containing three tyrosine residues. CD226 is in the immunoglobulin superfamily and modulates NK cell functions by interacting with PVR, Nectin-2, CD96, and TIGIT.^84,199–203^ In murine models, downregulation of CD96 enhanced antitumor functions of CD226, thereby reducing lung metastases and tumor growth. These findings indicate that CD226 has potential as an activating checkpoint receptor for immunotherapy.

NKG2D is an activating immune receptor expressed on NK cells that can trigger cytotoxicity and is in the CD94/NKG2 family of C-type lectin-like receptors.^200,204^ NKG2D binds to its ligands that occur on the surface of tumor cells, such as stem-like tumor cells, and alters these cells to be more susceptible to immune destruction.^205,206^ Alternatively, tumor cells can evade immune surveillance by shedding soluble NKG2D ligands. Recent studies showed that NKG2D targeting might be a practical immunotherapeutic approach for the treatment of cancer.^203,207,208^ Inducing activating receptors and their ligands can be targeted by some potential strategies such as NKG2D ligand α3 domain-specific antibodies, cytokines (IL-2, IL-15, and IL-21), histamine, anti-TGF-β monoclonal antibodies, TGF-β receptor I kinase inhibitors, TGF-β antisense oligonucleotides demethylating agents (5-azacytidine and 5-aza-2’-deoxycytidine) (Fig. 3).^209–216^ In particular, targeting the NKG2D/soluble MIC, which is highly expressed in many malignant carcinoma cells (like melanoma and leukemia), maybe a potential approach for the treatment of these cancers.

## NK cell as a promising therapeutic target for cancer

NK cells belong to the innate immune system and are active in the early fight against cancer through their ability to kill abnormal cells.^101,217^ They do not require previous stimulation and can induce an effective antitumor response that is mainly mediated by specialized cell-surface receptors, indicating malignancy.^218^ There is evidence from preclinical experiments and clinical trials for the development of NK cell-targeted immunotherapies (Tables 1 and 2) as a solution to chemo-resistant cancers, such as non-Hodgkin lymphoma.^80,219^ Monoclonal antibodies (mAbs) have been shown to elicit or augment prevailing antitumor immune responses. Their activity is referred to as ‘checkpoint blockade mAbs’ and is based on the principle of disturbing suppressive signals made by inhibitory receptors of lymphocytes. Recent methodologies have also been developed using the mAb-mediated blockade of definite NK cell immune checkpoints.^48,52,74,157,220^

**Table 1:** Preclinical experiments in promising cancer target of immune checkpoints

Immune checkpoint receptors	Treatment	Ligands	Signaling motif	Receptor adapter	Key results
Stimulating family					
NKG2D	NKG2D ligand α3 domain-specific antibodies, anti-NKG2A protein expression blockers (PEBLs)	MICA, MICB, and the ULBP family	YINM	DAP-10, DAP-12	The α3 domain-specific MICA/B antibody stimulated NK cells to produce IFN-γ and TNF-α^209^
KIR2DS/KIR3DS	Transfection of KIR2DS and DAP-12	classical HLA class I	ITAM	DAP-12	DAP-12 expression enhanced surface expression on NK cells and stability of KIR2DS^225^
Inhibitory family					
KIRs	Transfection of KIR-Fc GL183, EB6, DF200 and Pan2D (NKVSF1)	classical HLA class I	ITIM		KIR and HLA class I ligand interactions modulate NK cell immunity and antitumor activity^25,22,228^
TIGIT	13G6, SHIP-1 silencing, Y225A mutation	CD155, CD112, CD113	ITIM/ITT		Blockade of TIGIT signaling prevents NK cell exhaustion and enhances potent antitumor immunity^92,97,105^
CD96	3.3 mAb, 6A6 and 8B10	CD155	ITIM/YXXM		Anti-CD96 mAbs promote NK cell anti-metastatic activity in CD115-dependent and CD115-independent manner^120^
LAG-3	mAbs blocking FGL1/LAG-3 binding	MHC class II molecules, Fibrinogen-like Protein 1	KIEELE		mAbs blocking FGL1/LAG-3 binding stimulates tumor immunity^181^
NKG2A	anti-NKG2A PEBLs	HLA-E	ITIM		Anti-NKG2A PEBL transduction inhibited NKG2A expression without affecting NK cell proliferation, and generated more potent cytotoxicity than an anti-NKG2A antibody. The NKG2A^null^ NK cells increased NK cell killing of HLA-E–expressing tumors in vivo^43^
TIM-3	mAbs blocking TIM3-Fc binding to Gal-9, phosphatidylserine, Ceacam-1	Gal-9, phosphatidylserine, HMGB1, Ceacam-1	Tyrosine		Anti-Tim-3 antibodies inhibited Tim-3 signaling to improve the proliferation and cytotoxic activity of CD8^+^ TILs and reduce tumor-promoting cytokines production^146,147^

**Table 2:** Clinical trials targeting NK cell-inhibitory checkpoints in cancer treatment

Target	Agent	Clinical trials number	Phase	Status	Combination immunotherapy	Combination immunotherapy target	Tumor
TIGIT	MTIG7192A	NCT02794571	I	Recruiting	Atezolizumab	PD-L1	Advanced/metastatic tumors
		NCT03563716	II	Active, not recruiting	Atezolizumab	PD-L1	NSCLC
	BMS-986207	NCT02913313	I/II	Active, not recruiting	Nivolumab	PD-1	Broad solid tumor
	MK-7684-001	NCT02964013	I	Recruiting	Pembrolizumab	PD-1	Advanced solid tumors
	AB154	NCT03628677	I	Recruiting	Zimberelimab	PD-1	Advanced solid tumor
	ASP8374	NCT03260322	I	Recruiting	Pembrolizumab	PD-1	Advanced solid tumors
	TrasGEX™	NCT01409343	I	Completed			Solid tumors
LAG-3	Relatlimab	NCT03642067	II	Recruiting	Nivolumab	PD-1	MSS colorectal adenocarcinomas, colorectal adenocarcinoma
		NCT03743766	II	Recruiting	Nivolumab	PD-1	Metastatic melanoma
		NCT03607890	II	Recruiting	Nivolumab	PD-1	Refractory MSI-H solid tumors prior of PD-(L)1 therapy, MSI-H tumors
		NCT03724968	II	Active, not recruiting	Nivolumab, Ipilimumab	PD-1, CTLA-4	Metastatic melanoma stratified by MHC-II expression
		NCT03044613	I	Recruiting	Nivolumab	PD-1	Gastric cancer, esophageal cancer, GEJ cancer
		NCT03459222	I/II	Recruiting	Nivolumab, Ipilimumab, BMS-986205	PD-1,CTLA-4, IDO1	Advanced cancer
		NCT01968109	I/II	Recruiting	Nivolumab, BMS-986213	PD-1	NSCLC, gastric cancer, HCC, RCC, bladder cancer, SCCHN, melanoma
		NCT02966548	I	Recruiting	Nivolumab	PD-1	Advanced solid tumors
		NCT02996110	II	Recruiting	Ipilimumab, Nivolumab, BMS-986205	CTLA-4,PD-1,IDO1	Advanced RCC
		NCT02935634	II	Recruiting	Ipilimumab, Nivolumab, BMS-986205	CTLA-4,PD-1,IDO1	Advanced gastric cancer
		NCT02750514	II	Active, not recruiting	Ipilimumab, Nivolumab, BMS-986205, Dasatinib	CTLA-4,PD-1,IDO1,LAG3	Advanced NSCLC
		NCT03335540	I	Recruiting	Nivolumab, Cabiralizumab, Ipilimumab, IDO1 Inhibitor	PD-1,CSF-1R, CTLA-4,IDO1, KIR2DL1/2/3	Advanced cancer
		NCT03704077	II	Withdrawn	Nivolumab	PD-1	Gastric cancer, GEJ adenocarcinoma
		NCT03623854	II	Recruiting	Nivolumab	PD-1	Advanced chordoma
		NCT03470922	II/III	Recruiting	Nivolumab	PD-1	Advanced melanoma
	IMP321	NCT02676869	I	Completed	Pembrolizumab	PD-1	Stage IV or III melanoma
		NCT02614833	II	Active, not recruiting			Adenocarcinoma breast stage IV
		NCT03252938	I	Active, not recruiting	Avelumab	PD-L1	Solid tumors, peritoneal carcinomatosis
		NCT03625323	II	Recruiting	Pembrolizumab	PD-1	NSCLC, SCCHN
		NCT00349934	I	Completed			Metastatic breast cancer
		NCT00351949	I	Completed			Stage IV RCC
	XmAb®22841	NCT03849469	I	Recruiting	Pembrolizumab (Keytruda®)		Melanoma, Cervical carcinoma, Pancreatic carcinoma, TNBC, HCC, Urothelial carcinoma, SCCHN, Nasopharyngeal carcinoma, RCC, NSCLC, SCLC, Gastric or GEJ adenocarcinoma, Advanced or metastatic solid tumors, Prostate carcinoma, MSI-H, Mismatch repair deficiency, Epithelial ovarian cancer, Fallopian tube cancer, Primary peritoneal carcinoma, Intrahepatic cholangiocarcinoma
	TSR-033	NCT03250832	I	Recruiting	Dostarlimab	PD-1	Advanced solid tumors
	Sym022	NCT03489369	I	Completed			Metastatic cancer, Solid tumor, lymphoma
	REGN3767	NCT03005782	I	Recruiting	Cemiplimab (REGN2810)	PD-1	Advanced malignancies, including lymphoma
	MK4280	NCT03598608	I/II	Recruiting	Pembrolizumab		Hodgkin disease, non-Hodgkin lymphoma, B-cell lymphoma
		NCT02720068	I	Recruiting	Pembrolizumab	PD-1	Advanced solid tumors
	MGD013	NCT03219268	I	Recruiting			Advanced solid tumors, Hematologic neoplasms, Gastric Cancer, Ovarian cancer, GEJ cancer, HER2-positive breast cancer, HER2-positive gastric cancer, DLBCL
	LAG525	NCT03365791	II	Active, not recruiting	PDR001	PD-1	SCLC, Gastric adenocarcinoma, Esophageal adenocarcinoma, Castration-resistant prostate adenocarcinoma, Soft tissue sarcoma, Ovarian adenocarcinoma, Advanced well-differentiated neuroendocrine tumors, DLBCL
		NCT02460224	I/II	Active, not recruiting	PDR001	PD-1	Advanced solid tumors
		NCT03499899	II	Active, not recruiting	PDR001	PD-1	Advanced TNBC
		NCT03742349	I	Recruiting	Spartalizumab (PDR001), NIR178, MCS110,	PD-1, Adenosine A2A receptor, M-CSF	TNBC
	INCAGN0 2385	NCT03538028	I	Recruiting			Cervical cancer, MSI-H endometrial cancer, Gastric cancer (including GEJ), Esophageal cancer, HCC, Melanoma (uveal melanoma excluded), Merkel cell carcinoma, Mesothelioma, MSI-H colorectal cancer, NSCLC, Ovarian cancer, SCCHN, SCLC, RCC, TNBC, Urothelial carcinoma, DLBCL
	FS118	NCT03440437	I	Active, not recruiting			Advanced cancer, Metastatic cancer
	BI 754111	NCT03780725	I	Recruiting	BI 754091	PD-1	NSCLC, Head and neck neoplasms
		NCT03156114	I	Recruiting	BI 754091	PD-1	Advanced solid tumors, NSCLC
		NCT03433898	Early Phase I	Recruiting	BI 754091	PD-1	Advanced solid tumors
		NCT03697304	II	Suspended	BI 754091	PD-1	Advanced and/or Metastatic solid tumors
NKG2A	Monalizumab	NCT02921685	I	Recruiting			Hematologic malignancies
		NCT02671435	I/II	Active, not recruiting	Durvalumab	PD-L1	Advanced solid tumors
		NCT02643550	I/II	Recruiting	Anti-PD(L)1	PD(L)1	Recurrent or metastatic SCCHN
		NCT02459301	I	Completed			Gynecologic cancer
TIM-3	TSR022	NCT02817633	I	Recruiting	Nivolumab, TSR-042, TSR-033	PD-1, LAG-3	Advanced or metastatic solid tumors
		NCT03680508	II	Recruiting	TSR-042	PD-1	Locally advanced or metastatic liver cancer
	Sym023	NCT03489343	I	Active, not recruiting			Metastatic cancer, solid tumor, lymphoma
		NCT03311412	I	Recruiting	Sym021, Sym022	PD-1, LAG-3	Metastatic cancer, solid tumor, lymphoma
	SHR-1702	NCT03871855	I	Not yet recruiting	Camrelizumab	PD-1	Advanced solid tumor
	RO7121661	NCT03708328	I	Recruiting			Solid tumors, Metastatic melanoma, NSCLC, SCLC, Esophageal squamous cell carcinoma
	MBG453	NCT03066648	I	Recruiting	PDR001	PD-1	AML, high risk MDS
		NCT02608268	I/II	Recruiting	PDR001	PD-1	Advanced solid tumor
	LY3321367	NCT03099109	I	Active, not recruiting	LY3300054	PD-L1	Advanced relapsed/refractory solid tumor
	INCAGN2390	NCT03652077	I	Recruiting			Cervical cancer, Gastric cancer, GEJ cancer, Esophageal cancer, HCC, Melanoma, Uveal melanoma, Merkel cell carcinoma, Mesothelioma, MSI, NSCLC, Ovarian cancer, SCCHN, SCLC, RCC, TNBC, Urothelial carcinoma, Mismatch repair deficiency
	BMS-986258	NCT03446040	I/II	Recruiting	Nivolumab	PD-1	Advanced cancer
	BGB-A425	NCT03744468	I/II	Recruiting	Tislelizumab	PD-1	Locally advanced or metastatic solid tumors
NKG2D	CYAD-101(NKR-2)	NCT03692429	I	Recruiting			Colorectal cancer
		NCT03466320	I/II	Recruiting			AML, Adult MDS
		NCT03370198	I	Active, not recruiting			Colon cancer liver metastasis
		NCT03310008	I	Active, not recruiting			Colon cancer liver metastasis
		NCT03018405	I/II	Recruiting			AML, MDS, Multiple myeloma

*MSS* microsatellite stable, *NSCLC* non-small cell lung cancer, *MSI-H* microsatellite instability high, *GEJ* gastroesophageal junction, *SCLC* small cell lung cancer, *SCCHN* squamous cell carcinoma of the head and neck, *DLBCL* diffuse large B-cell lymphoma, *HCC* hepatocellular carcinoma, *RCC* renal cell carcinoma, *TNBC* triple-negative breast cancer, *AML* acute myeloid leukemia, *MDS* myelodysplastic syndromes

The restoration of NK cell activity against HLA-I^+^ tumor cells is essential in the therapeutic targeting for novel immunotherapies that depend on therapeutic monoclonal antibodies, such as anti-pan-KIR2D.^67,221,222^ In the absence of infection, inhibitory HLA-KIR signals dominate and protect tumor cells from NK cell-mediated lysis, while intracytoplasmic binding of KIR2DL or KIR3DL with ITIMs prevent NK cell lysis activity.^68,223^ Conversely, KIR2DS and KIR3DS are noncovalently associated with an ITAM–bearing adapter molecule DAP-12, which results in phosphorylation of tyrosine residue in the ITAM and recruiting ZAP-70 or Syk, leading to cellular activation and increases the capacity of NK cells to recognize tumor cells; this is an essential target for cancer treatment.^26,224,225^ KIR has been established to play a critical role in mediating self-tolerance along with the facilitation of cytotoxicity against infected cells and transformed cells. According to research, KIR-HLA relationships can be linked to the incidence and course of both solid tumors and hematologic malignancies.^67,226^ The inhibitory KIR-HLA relationships have been established as being overrepresented in patients with acute and chronic leukemia, breast cancer, Hodgkin lymphoma, and melanoma.^226^ The assertion can be understood based on matched autologous inhibitory KIR-HLA interactions preventing the lysis of cancerous target cells.^227^ Autologous NK cell therapy, which is a novel expansion method and will help patients at the advanced stage of digestive cancer, is in phase I trial.^227^ As part of targeting cancer therapies, the expression of activating KIRs in different circumstances can be linked to better outcomes in some malignancies.^26^ KIR-ligand modulation is vital for cancer therapy as exemplified by the initial therapeutic manipulation of KIR-ligand relationships in the case of T cell-depleted, haploidentical hematopoietic stem cell transplantation for hematologic malignancies. The inhibitory KIR-ligand relationship has been established as important in the realization of NK cell alloreactive potential with special attention on the balance of activating and inhibitory signals that direct cytotoxicity.^26,228^

Preclinical studies and clinical trials of PD-1 blockade therapy have led to impressive results in mediating tumor eradication. As discussed, NK cells secrete pro-inflammatory cytokines that promote the expression of PD-L1 in tumor cells and enhance the inhibiting role of PD-1. At the same time, PD-1 antibodies can bind to NK cell surface PD-1, prevent NK cell depletion, and enhance NK cell antitumor response.^229,230^ Pidilizumab (previously CT-011) is an anti-PD-1 antibody that recently entered clinical trials. Pidilizumab can enhance human NK cell activity against autologous primary multiple myeloma cells.^4,231–233^ In addition, highly dense activated human primary NK cells can kill colorectal carcinoma cells grown in 3D cultures independent of PD-L1 expression, suggesting that the use of allogeneic activated NK cells could be an effective treatment of this cancer.^38,77,93,234–236^ Immune evasion *via* PD-1/PD-L1 in NK cells and monocytes/macrophages is prominent in Hodgkin’s lymphoma.^38,77,93,234–237^ Also, one of the immune checkpoint molecules that have already been employed in specific preclinical tumor models is the establishment of efficacy in blocking TIGIT to modulate NK cell function and has resulted in improved clinical responses in patients with cancer.^7,94,95^

There are ongoing phase I/II trials of the application of NK cells combined with PD-1 antibodies (sintilimab and pembrolizumab) in the treatment of patients with advanced non-small-cell lung cancer (NCT03958097) and advanced biliary tract cancer (NCT03937895). Thus, there are many ongoing studies of immunotherapies for PD-1 blockade by targeting NK cells in cancer treatment.

Lirilumab targeting pan-KIR2D and monalizumab targeting NKG2A have been demonstrated to disrupt the interactions of inhibitory KIR on NK cells with classical HLA class I-peptide complex on tumor cells or NKG2A with nonclassical HLA-I molecular HLA-E, which could unleash the antitumor NK cell cytotoxic activity mimicking “missing-self” response.^16,69,238^ Both lirilumab and monalizumab are safe with limited side effects upon prolonged treatment in phase I clinical trials.^237^ Markedly, all dosages of lirilumab induced high levels of free KIR2Ds (>95%).^237^ These agents are currently undergoing phase I/II clinical trials across a range of hematologic and solid tumors in monotherapy or combination with other immune checkpoint blockades, including PD-1 inhibition (NCT02671435 and NCT02643550).^239^

The role of NK cells in cancer therapy also promotes the activating receptor CD226, which is a mediator of the cells’ responses against tumors. Although, the mechanism by which the CD226 receptors exert control over the function of NK cell is a challenge, the engagement of the receptor with CD155 in the transcription factor FOXO1 phosphorylation, which results in the inactivation of the receptor’s negative regulatory control over the effector function of NK cell.^226,240^

As discussed, the activated NKG2D receptor is a critical NKG2 family receptor on NK cells and can bind to NKG2D ligands expressed on tumor cells. This binding allows NK cells to activate and destroy tumor cells, as well as produce cytokines. The NKG2D/NKG2D ligand axis has been recognized to play an essential role in antitumor activity. Infusions of activated NK cells can scavenge circulating soluble MICA in cancer patients, leading to reinstate NKG2D-mediated immune surveillance.^235^ Although our understanding of the control of NKG2D ligand expression remains limited, research in recent years revealed various cellular mechanisms by which cancer cells evaded detection by reducing the “stress-induced ligands” expression. Recently, NKG2D ligand (MICA and MICB) α3 domain-specific antibodies were developed to restore NK cell-mediated tumor immunity by increasing the density of stimulatory MICA and MICB ligands on the surface of tumor cells along with reducing the shedding of both MICA and MICB.^236^ Therefore, targeting co-stimulatory receptor signaling may serve as an alternative strategy to boost NK cell function and prove beneficial for cancer treatment (Fig. 3).

The difference between the current treatment strategies and the promising immune checkpoint molecules such as TIGIT in NK cells is that they can be modulated to enhance clinical responses. The diverse receptors expressed on the tumor-infiltrating surfaces of NK cells would increase the binding of tumors once activated and destroy them.^121^ The establishment of the receptors, especially the IL-1R2 and IL-1R8, which are effective immune regulators, will improve immune surveillance and control.^165^ Single immunoglobulin IL-1R-related receptor, SIGIRR, also known as TIR8 or IL-1R8, PD-1, LAG-3, CTLA4, and TIM-3 has distinct functional and structural characteristics that adapt it better to regulate the expression of NK cells in a TME.^13,241^ Other receptors that can complement the role of NK cell subsets to increase their prediction of malignancy are TIM-3 and IL-15.^154,242^

Given different cancer types and tumor staging, it is hard to say which is better for restoring NK cell-mediated tumor immunity between targeting the NKG2D receptor and targeting KIR or NKG2A. Although one recent in vitro study found blocking the inhibitory signal (blocking of HLA–KIR) is not so efficient as the activating signal increases (Ara-C treatment that induced NKG2D ligands) potentiate NK cell responses against acute myelocytic leukemia (AML) cells, more in vitro and in vivo studies are needed to demonstrate it. However, this study provides evidence that a combination of both treatments has better efficiency.^243^

Recently, adoptive cell therapy strategies, including cancer-targeting CARs-engineered NK cell infusion have been explored in preclinical and clinical studies.^169–173^ Chang et al. demonstrated a chimeric receptor with the NK cell-activating molecule NKG2D, plus two vital signaling molecules, DAP-10 and CD3ζ, enhanced the antitumor effect of peripheral blood NK cells on osteosarcoma.^169^ More recently, human induced pluripotent stem cells (iPSCs) have been proven to be more efficient in genetically modifying and expressing CAR constructs specifically designed to enhance NK cell antitumor activity.^244^ To date, CAR engineered NK cells, which are from autogenic or allogeneic peripheral blood mononuclear cell, umbilical cord blood, human embryonic stem cell, human iPSC, and NK cell lines,^244–254^ are at the beginning but greatly promising in tumor immunotherapy.

The increasing resistance of several cancers to chemotherapy demands a clinical strategy to increase immune checkpoint molecules’ effectiveness. Findings from a study indicated that the elimination of regulatory T cells (Treg) using IL-2–diphtheria fusion would have a therapeutic benefit if haploidentical NK cells are infused in AML patients. The study shows that Treg cells have a negative role in population expansion of NK cells following transfer. In this first clinical trial, an administration of recombinant IL-15 clearing lung cancer lesions in metastatic cancer patients astatic further supports the effectiveness of immune checkpoint molecules in NK cells as target cancer immunotherapy.^255^ Combined therapy is emerging more effective strategy for treating cancer. Although targeting NK cells is a leading intervention cancer immunotherapy through their direct lysis of tumor cells, the intervention’s effectiveness is reduced by immune evasion.^256–258^ Combination therapy provides an alternative strategy for increasing the effectiveness of NK cells’ antitumor ability.^55^ One of the target combinations is NK cells with nanomaterials and oncolytic viruses. TIGIT combined with CD112 and CD155; blocking CD96 is considered another promising therapeutic role.^259^ Research findings have proved that a combination of TIGIT blockade and anti-PD-1/PD-L1 antibodies forms a synergy that may be used in preclinical trials.^156^ Other findings from a recent study indicated that the checkpoint receptor TIGIT blockage prevents the exhaustion of NK cell and triggers the elicitation of T-cell immunity that are potent tumor-specific in a NK cell-dependent manner.^19,260^ A combination of engineering NK cells with specific immune checkpoint signaling domains also provides a promising therapeutic potential for the treatment of recurrent and refractory cancer.

## Study and progress of adenosine in the NK cell cancer immunotherapy

To complement the general role of control and surveillance of malignant cells that the immune system play, the development of adoptive cellular therapy and checkpoint blockade has brought about a revolution in the landscape of cancer therapy and promises the potential in which oncologists can utilize a patient’s immune system to treat cancer.^185,261–263^ Purinergic signaling axis has gained prominence as one of the mechanisms of tumor-mediated immunosuppressions, in which the purine nucleoside adenosine produced in the TME has the ability of potently suppressing NK cell function.^264–266^ During this event, the production of extracellular adenosine is triggered by the mediation of the cell surface ectoenzymes CD38, CD39, and CD73,^267–269^ and the development of therapeutic agents has focused on targeting these enzymes as responses.^270^ The therapeutic agents also target receptors, including the downstream adenosine receptors (AR) such as A_1_R, A_2B_R, A_2A_R, and A_3_R, to promote antitumor immune responses.^16,264,271^

In NK cells, the signaling of adenosine (ADO) signals via A_2A_R suppresses their cytokine production and cytotoxicity function.^272,273^ ADO signaling plays a critical role in immune regulation, as further underscored by the NK cells total dysfunction in individuals presenting with a variant of severe combined immunodeficiency caused when adenosine deaminase (ADA) undergoes a mutation that catalyzes ADO conversion to inosine.^262,274,275^ Also, the expression of CD38 on tumors, T cells, and NK cells promote the generation of ADO followed by proliferation and suppression of T cell function. In addition to suppressing T cell responses, cAMP has an overall inhibitory effect on NK cells.^264,276–278^

**Fig. 4:**
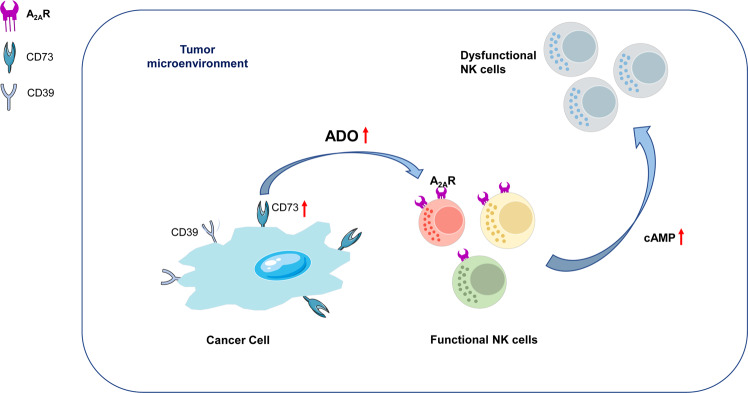
Adenosine-axis in the tumor microenvironment mediates immune suppression of NK cells. In the tumor microenvironment, stress such as hypoxia increases extracellular ATP levels and CD73 expression on cancer cells, leading to adenosine (ADO) upregulation and the activation of downstream signaling through A_2A_R on NK cells. ADO/A_2A_R signaling results in dysfunction of NK cell metabolic and effector functions

NK cells express high A_2A_R levels, specific A_2A_R agonists, or ADO suppressed NK cells due to their cytokine production and cytotoxic function.^142,279–281^ Intracellular cAMP concentration enhancement mediated by A_2A_R is believed to be the predominant mechanism explaining how ADO suppresses NK cell activity (Fig. 4).^264,282,283^ Like T cells, NK cells express the A_3_R, the function of which has been found to regulate A_3_R positively and NK cells, resulting in metastatic and primary tumor growth both in human and mouse models of melanoma and colon cancer.^284^

There are several trials on approaches involving a combination of CD38 with anti-PD-1 that have been found to stimulate enhanced antitumor responses by T cell-mediated by enhanced Granzyme B and IFN-γ expression by CD8^+^ T cells.^79,264,285^ Findings from another study showed that A_2A_R cells had enhanced the penetration in hypoxic tumors.^286^ Although the focus of most of the combination approaches indicates the possibility of ADO targeting aimed at enhancing T cell responses, ADO axis targeting, including NK cells, can enhance other immune subsets comprised in the TME.^287^ The accumulation of ADO predominantly in the TME when the extracellular ATP is catabolized to ADO by CD38, CD39, and CD73 is expressed in immune and tumor cells, and it has been reported that ADO mediates the suppression of antitumor immunity when the ADO receptors.^185,268^

### Extracellular ADO production in the TME

TME forms site for high ADO concentrations caused by inflammation, tissue disruption, and contribution of stromal and immune cells.^288,289^ Hypoxia is another predominant driver of increased ADO concentration through stimulation of large ATP formation.^290^ It also contributes to the expression of HIF-1α, a well-defined transcription factor, which stimulates the expression of ectoenzymes CD73 (5’-NT) and CD39 (NTPDase1) on stromal cells, tumor cells, as well as tumor-infiltrating immunosuppressive cell subsets, for example, myeloid-derived suppressor cells and Treg cells.^242,291^ Part of the evidence to support the role of CD39 and CD73 in tumorigenesis and inflammation is that mice deficient in CD73 or CD39 are susceptible to autoimmunity/inflammation and be tumor resistant as a result of ADO-mediated immunosuppression alleviation.^292–294^

### Targeting the adenosine pathway in TME to improve immunotherapies

Cancer immunotherapies are currently considered the fourth pillar in the treatment of cancer.^295^ Several research studies on the role of ADO negatively regulating NK cell responses through A_2A_R, and naturally targeting the ADO pathway are part of cancer immunotherapy interventions that may further boost the efficacy of approved immunotherapies in the clinic.^35,267,296,297^ Although ADO has been found to thwart the antitumor immune responses that radiotherapy elicits, when ADO receptors are antagonized in cancers, the approach allows the interruption of the ADO-dependent immune evasion that results in antitumor immune responses.^298^ The immunosuppressive role of NK cells, combination strategies rationally targeting the pathway with adoptive cell therapies (ACT), and checkpoint inhibitors can synergistically promote the function of antitumor immune cell function, a process that utilizes ATP generated in the TME (Fig. 4) (Table 3).^271,299,300^

**Table 3:** Clinical evaluation targeting the adenosine pathway

Molecular target	Clinical trial number	Study phase	Cancer type	Agents	Combination
A_2A_R	NCT03207867	II	Solid tumors and non-hodgkin lymphoma	NIR178	PDR001 (anti-PD-1)
A_2A_R	NCT02403193	I/II	Non-small cell lung cancer (NSCLC)	PBF-509	PDR001 (anti-PD-1)
A_2A_R	NCT02655822	I	Advanced cancers	Ciforadenant	Atezolizumab (anti-PD-L1)
A_2A_R	NCT04266665	IV	Glioma	Dexmedetomidine	Craniotomy
A_2A_R	NCT04280328	I	Relapsed or refractory multiple myeloma	CPI-444	Daratumumab (anti-CD38)
A_2A_R and A_2B_R	NCT04262856	II	Non-small cell lung cancer (ARC-7)	AB928	Zimberelimab (anti-PD-1), AB154 (anti-TIGIT)
A_2A_R and A_2B_R	NCT03629756	I	Advanced malignancies	AB928	AB122 (anti-PD-1)
A_2A_R and A_2B_R	NCT03719326	I	Triple-negative breast cancer or gynecologic malignancies	AB928	Multiple drug combinations
A_2A_R and A_2B_R	NCT03846310	I	NSCLC	AB928	Zimberelimab (anti-PD-1), Pembrolizumab (anti-PD-1), Carboplatin, Pemetrexed
A_2A_R and A_2B_R	NCT03720678	I	Advanced metastatic gastroesophageal Cancer (GEC) or colorectal cancer (CRC)	AB928	mFOLFOX
A_2A_R, A_2B_R, and CD73	NCT04381832	I/II	Metastatic castrate-resistant prostate cancer (mCRPC)	AB928, AB680	Zimberelimab (anti-PD-1), Enzalutamide (androgen receptor inhibitor), Docetaxel
A_2A_R, CD73	NCT02111330	I	NSCLC	AZD4635, MEDI9447	Osimertinib (EGFR Kinase inhibitor)
A_2A_R, CD73	NCT04089553	II	Prostate cancer	AZD4635, Oleclumab	Durvalumab (anti-PD-1)
A_2A_R, CD73	NCT03549000	I	Advanced cancers	NIR178, NZV930	PDR001 (anti-PD-1)
A_2B_R	NCT03274479	I	NSCLC	PBF-1129	

## Limitations of immune checkpoint molecules in NK cell for cancer immunotherapy

Age is the main contributor to NK cell altered expression of checkpoint molecules in NK cells.^47,301^ Activating receptors alteration has been associated with chronic exposure of the receptors to ligands on tumor cells.^302^ There is also a synergistic effect between age and cancer that diminishes NK cell-mediated tumor immunosurveillance, which may also be associated with NK cell immunoresistance.^257,303^ However, oncologists have made significant successes in developing different strategies to harness the power of NK cells aimed at targeting tumor cells, such as adoptive transfer therapy, which involves allogeneic or autologous expanded NK cells.^2,304^

## Future directions to study the NK cells in cancer immunotherapy

Although numerous trials have been carried out on various innovations on cancer therapy, cancer treatment is still a significant challenge. Proposals by academia, such as identifying allogeneic NK cells as one of the most effective cancer treatment options, have not made much progress in identifying a cancer therapy applicable to a majority of cancers without issues such as NK cell receptor downregulation.^305^ In my opinion, leaving these studies the academia and biotech companies would not bring a solution any time soon. I propose that the World Health Organization developed governments and other non-governmental organization fund multi-disciplinary groups and organizations in the healthcare field to conduct researches aimed at both preventive and curative recommendations for cancer therapy, especially for stage I to III treatment strategies for non‐small cell lung cancer, in which radiotherapy and surgery are usually employed.^306^ Therefore, considering combinatorial approaches drawing together different treatment strategies that involve NK cell functions would be worthwhile. NK cell research companies should consider developing NK cell-specific treatments underlying scientific findings and principles of their best product pipelines, demonstrating highly innovative concepts and practices that herald future sustainable and cost-effective clinical applications.

Since the role of NK cell-based immunotherapy strategies has gained immense support as a proven intervention against cancer, I advise that everyone should not consider it as the bottom-line solution based on which all future research will focus. NK cells can only be effective in a few types of cancer, considering their complex networking, heterogeneity, as well as the inherent adaptability of a wide range of tumors that evade destruction by immune cells. In the meantime, future studies should focus resources on improving the efficacy of NK cell products that are currently available.^94^ The discoveries of all NK cell-based immunotherapies should proceed to clinical implementation.^306^

## Conclusion

Human NK cells are essential and play a significant role in the development of therapeutic treatments for cancer. In the tumor microenvironment, NK cells transform to become dysfunctional cells with enhanced expression of inhibitory immune checkpoints, including the non-HLA-class I-specific inhibitory receptors (such as PD-1, TIGIT, CD112R, CD96, IL-1R8, and TIM-3) and HLA-class I-specific inhibitory receptors (such as KIR, NKG2A, and LAG-3), as well as decreased expression of activating receptors (such as NKG2D and CD226). Adenosine-A_2A_R signaling is emerging as a novel inhibitory immune checkpoint pathway in NK cells. Blockade of these inhibitory checkpoint molecules with the use of immune receptor inhibitors, as well as activation of co-stimulatory receptor signaling by inducing co-stimulatory receptor expression, can reinstate the antitumor activity of NK cells. Many primary researches support the efficacy of NK cell-targeted immunotherapies based on immune checkpoint molecules in NK cells at preclinical phase and are ongoing at different clinical phases. In addition, more research is needed on the safety, tolerability, and clinical efficacy of drugs targeting these checkpoints and their combination therapy. There is a need to identify highly effective strategies of overcoming immune resistance, the main challenge in cancer immunotherapy, such as through CAR engineered NK cells. Above all, an in-depth understanding of immune checkpoint molecules that target NK cells could help develop new therapeutic strategies against refractory cancers.

## Supplementary information

Table 2 (continued)
