# Supplementary material for: Immune checkpoint molecules in natural killer cells as potential targets for cancer immunotherapy
Source: Signal Transduct Target Ther. 2020 Oct 29;5:250. doi: 10.1038/s41392-020-00348-8 (PMC7596531; doi:10.1038/s41392-020-00348-8)
Supplement: Supplementary file 1 — Table 2 (continued) [file 41392_2020_348_MOESM1_ESM.docx]

**Table 2. Clinical trials targeting NK cell-inhibitory checkpoints in cancer treatment (continued)**

| **Clinical trials**  **number** | **Study title** | **Efficacy** | **Adverse Events**  **(at risk %)** |
| --- | --- | --- | --- |
| NCT02794571 | Safety and Pharmacokinetics (PK) of Escalating Doses of MTIG7192A as a Single Agent and in Combination With Atezolizumab With and Without Chemotherapy in Locally Advanced or Metastatic Tumors | No results posted | No results posted |
| NCT03563716 | A Study of MTIG7192A in Combination With Atezolizumab in Chemotherapy-Naïve Patients With Locally Advanced or Metastatic Non-Small Cell Lung Cancer | 135 pts randomly assigned to MTIG7192A plus atezolizumab maintained the ORR and progression-free survival benefits. | Placebo + Atezolizumab (35.29%)  MTIG7192A + Atezolizumab (34.33%) |
| NCT02913313 | An Investigational Immuno-therapy Study to Evaluate the Safety and Effectiveness of Experimental Medication BMS-986207 by Itself and in Combination With Nivolumab in Solid Cancers That Are Advanced or Have Spread | No results posted | No results posted |
| NCT02964013 | Study of Vibostolimab Alone and in Combination With Pembrolizumab in Advanced Solid Tumors (MK-7684-001) | DCR in MK-7684 monotherapy (35%) and MK7684 plus pembrolizumab (47%) | Adverse events (AEs) occurred in 53% of monotherapy and 65% of combination therapy recipients  (grade 3-5, 6% and 12%). |
| NCT03628677 | A Study to Evaluate the Safety and Tolerability of AB154 in Participants With Advanced Malignancies | No results posted | No results posted |
| NCT03260322 | A Multiple-dose Study of ASP8374, an Immune Checkpoint Inhibitor, as a Single Agent and in Combination With Pembrolizumab in Subjects With Advanced Solid Tumors | No results posted | No results posted |
| NCT01409343 | TrasGEX™: Dose Escalation Study | The pharmacokinetic properties support a q3w infusion scheme. Evidence of activity was seen in 40.6% of pts. | No dose limiting toxicity was observed. Infusion-related reactions were the most frequently observed drug-related AEs (51.4%), all but two of grade 1 or 2. |
| NCT03642067 | Study of Nivolumab and Relatlimab in Patients With Microsatellite Stable (MSS) Advanced Colorectal Cancer | No results posted | No results posted |
| NCT03743766 | Nivolumab, BMS-936558 in Combination With Relatlimab, BMS-986016 in Patients With Metastatic Melanoma Naïve to Prior Immunotherapy in the Metastatic Setting | No results posted | No results posted |
| NCT03607890 | Study of Nivolumab and Relatlimab in Advanced Mismatch Repair Deficient Cancers Resistant to Prior PD-(L)1 Inhibitor | No results posted | No results posted |
| NCT03724968 | Nivolumab Plus Relatlimab or Ipilimumab in Metastatic Melanoma Stratified by MHC-II Expression | No results posted | No results posted |
| NCT03044613 | Nivolumab +/- Relatlimab Prior to Chemoradiation With II/III Gastro/Esophageal Cancer | Nivolumab +/- Relatlimab induced a pathologic complete response of 40% in stage II/III E/GEJ cancers. | Induction Nivolumab and neoadjuvant Nivolumab combined with neoadjuvant chemoradiation has an acceptable toxicity profile. Toxicities include steroid-responsive grade 3 dermatitis (1/16), grade 3 hepatitis (1/16), and no pneumonitis cases. |
| NCT03459222 | An Investigational Study of Immunotherapy Combinations in Participants With Solid Cancers That Are Advanced or Have Spread | No results posted | No results posted |
| NCT01968109 | An Investigational Immuno-therapy Study to Assess the Safety, Tolerability and Effectiveness of Anti-LAG-3 With and Without Anti-PD-1 in the Treatment of Solid Tumors | ORR was 16% (confirmed/unconfirmed), and DCR was 45% with benefit observed. | All grade and grade 3/4 treatment-related AEs occurred 46% and 9%, respectively, across all dose-expansion pts. |
| NCT02966548 | Safety Study of BMS-986016 With or Without Nivolumab in Patients With Advanced Solid Tumors | No results posted | No results posted |
| NCT02996110 | A Study to Test Combination Treatments in People With Advanced Renal Cell Carcinoma (FRACTION-RCC) | ORR was 15.2%; no pts achieved a complete response, and 7 achieved partial response. | Any-grade treatment-related AEs (78.3%); fatigue, rash [both 19.6%], and diarrhea [17.4%] were most common); Grade 3–4 treatment-related AEs (28.3%; diarrhea [8.7%], ↑amylase and ↑lipase [both 6.5%] were most common); Treatment-related immune-mediated AEs of any grade (47.8% ; rash [19.6%], diarrhea [17.4%], and ↑alanine aminotransferase [8.7%]). |
| NCT02935634 | A Study to Test Combination Treatments in Patients With Advanced Gastric Cancer (FRACTION-GC) | No results posted | No results posted |
| NCT02750514 | An Investigational Immuno-therapy Study to Test Combination Treatments in Patients With Advanced Non-Small Cell Lung Cancer (FRACTION-Lung) | No results posted | No results posted |
| NCT03335540 | An Adaptive Study to Match Patients With Solid Tumors to Various Immunotherapy Combinations Based Upon a Broad Biomarker Assessment (ADVISE) | No results posted | No results posted |
| NCT03704077 | An Investigational Immuno-therapy Study of Relatlimab Plus Nivolumab Compared to Various Standard-of-Care Therapies in Previously Treated Participants With Recurrent, Advanced or Metastatic Gastric Cancer or Gastroesophageal Junction Adenocarcinoma | No results posted | No results posted |
| NCT03623854 | Nivolumab and Relatlimab in Treating Participants With Advanced Chordoma | No results posted | No results posted |
| NCT03470922 | A Study of Relatlimab Plus Nivolumab Versus Nivolumab Alone in Participants With Advanced Melanoma | No results posted | No results posted |
| NCT02676869 | Phase 1 Study of IMP321 (Eftilagimod Alpha) Adjuvant to Anti-PD-1 Therapy in Unresectable or Metastatic Melanoma (TACTI-mel) | In 50 % of pts, a tumor reduction was observed. | No dose limiting toxicities or AEs ≥ grade 4 were reported. No patient discontinued treatment due to an AE. |
| NCT02614833 | IMP321 (Eftilagimod Alpha) as Adjunctive to a Standard Chemotherapy Paclitaxel Metastatic Breast Carcinoma | Increased circulating monocytes, dendritic cells, and CD8 T cells and increased cellular activation were observed. This sustained (≥ 6 months) activation of the cellular response was associated with increased plasma Th1 markers levels. Seven pts (47 %) had a partial response. The DCR was 87 %. | Nine (67 %) pts had a serious AE, out of which one was related to paclitaxel and one to efti (cytokine release syndrome grade 1). Grade 1 and 2 injection site reactions were the most common efti related AEs and occurred in 14 pts (93 %). |
| NCT03252938 | Feasibility and Safety of IMP321 for Advanced Stage Solid Tumors | Of the 8 pts enrolled so far, 4 had disease progression (acc. to RECIST 1.1), 1 partial response, 1 stable disease with some extent of tumor shrinkage, and 2 have not had tumor assessment yet. | The most common grade 1-2 AEs were pain, nausea, injection site reaction in 50%, 33%, and 17%. The most common grade 3 AEs were nausea/vomiting, preileus/ileus, ascites in 33%, 33%, and 17%. One AE grade 5 (acute kidney injury) was reported. 4 AEs grade 1-2 were possibly or related to IMP321 (injection site reaction 2x; fever; lipohypertrophy), 6 AEs grade 1-2 were possibly or related to avelumab (nausea 2x; chills; fever; dyspnea; lipohypertrophy). All AEs grade 3-5 were unrelated to any of the study drugs. |
| NCT03625323 | Combination Study With Soluble LAG-3 Fusion Protein Eftilagimod Alpha (IMP321) and Pembrolizumab in Patients With Previously Untreated Unresectable or Metastatic NSCLC, or Recurrent PD-X Refractory NSCLC or With Recurrent or Metastatic HNSCC (TACTI-002) | Eight pts (47 %) had a iPR and six (35 %) had stable disease according to iRECIST representing an ORR (DCR) of 47 % (82 %). irPRs were observed in all different PD-L1 groups ( < 1%; ≥ 1 % ≤49 %; ≥ 50 %). | The most common ( > 10%) AEs being cough (31 %), asthenia (23 %), decreased appetite (19 %), fatigue (19 %), dyspnea (17 %), diarrhea (15 %) and constipation 13 %). |
| NCT00349934 | IMP321 Plus First-line Paclitaxel in Metastatic Breast Carcinoma | No results posted | No results posted |
| NCT00351949 | IMP321 Phase 1 Trial in Metastatic Renal Cell Carcinoma (MRCC) | No results posted | No results posted |
| NCT03849469 | A Study of XmAb®22841 Monotherapy & in Combination w/ Pembrolizumab in Subjects w/ Selected Advanced Solid Tumors (DUET-4) | No results posted | No results posted |
| NCT03250832 | Study of TSR-033 With an Anti-programmed Cell Death-1 Receptor (PD-1) in Participants With Advanced Solid Tumors (CITRINO) | No results posted | No results posted |
| NCT03489369 | Sym022 (Anti-LAG-3) in Patients With Advanced Solid Tumor Malignancies or Lymphomas | No results posted | No results posted |
| NCT03005782 | Study of REGN3767 (Anti-LAG-3) With or Without REGN2810 (Anti-PD1) in Advanced Cancers | 2 (both small cell lung cancer) combo pts and 2 (endometrial cancer and cutaneous squamous cell carcinoma) of 12 additional pts who crossed over from monotherapy to combination therapy had partial responses. | No DLTs. The most common treatment-emergent AE was nausea (22.2%), fatigue (33.3%) and nausea (21.4%). Grade ≥3 immune-related adverse events (irAEs) were increased alanine and aspartate aminotransferases (each 3.7%) and hypothyroidism (2.4%). |
| NCT03598608 | Study to Evaluate the Safety and Efficacy of a Combination of MK-4280 and Pembrolizumab (MK-3475) in Participants With Hematologic Malignancies (MK-4280-003) | No results posted | No results posted |
| NCT02720068 | Study of MK-4280 as Monotherapy and in Combination With Pembrolizumab (MK-3475) With or Without Chemotherapy AND MK-4280A as Monotherapy in Adults With Advanced Solid Tumors (MK-4280-001) | ORR of MK-4280 (6%) and of in combination with  Pembrolizumab (27%) | AEs occurred in 61% of monotherapy and 53% of combination therapy  recipients were of grade 3-4 toxicity in 6% and 20%. |
| NCT03219268 | A Study of MGD013 in Patients With Unresectable or Metastatic Neoplasms | 3 of 41 cPRs per RECIST 1.1 were observed, while 21 pts had SD. In a cohort of pts with HER2+ tumors treated with MGD013 in combination with margetuximab, 3 PRs have been observed, and 2 pts with SD among 6 RE pts. Objective responses have been observed in several pts after prior anti-PD-1 therapy. | Treatment-related AEs occurred in 146/207 (70.5%) pts, most commonly fatigue (19%) and nausea (11%). The rate of Grade ≥ 3 AEs was 23.2%. |
| NCT03365791 | PDR001 Plus LAG525 for Patients With Advanced Solid and Hematologic Malignancies | No results posted | No results posted |
| NCT02460224 | Safety and Efficacy of LAG525 Single Agent and in Combination With PDR001 in Patients With Advanced Malignancies | LAG525 + spartalizumab led to durable RECIST responses (11 PR, 1 CR) in various solid tumors. In TNBC tumor biopsies, there was a trend in the conversion of immune-cold to immune-activated biomarker profiles. | Common (≥10%) related AEs were fatigue (10%) for LAG525 alone and fatigue (18%), diarrhea (15%), and nausea (12%) for the combination. Gr 3–4 related AEs were reported in 10 pts (8%) in the LAG525 arm and 10 pts (8%) in the combination arm. |
| NCT03499899 | A Study of Efficacy and Safety of LAG525 in Combination With Spartalizumab, or With Spartalizumab and Carboplatin, or With Carboplatin, in Patients With Advanced Triple-negative Breast Cancer | No results posted | No results posted |
| NCT03742349 | Study of Safety and Efficacy of Novel Immunotherapy Combinations in Patients With Triple Negative Breast Cancer (TNBC) | No results posted | No results posted |
| NCT03538028 | A Safety and Tolerability Study of INCAGN02385 in Select Advanced Malignancies | No results posted | No results posted |
| NCT03440437 | FS118 First in Human Study in Patients With Advanced Malignancies After PD-1/PD-L1 Containing Therapy | No results posted | No results posted |
| NCT03780725 | This Study Tests How BI 754111 is Distributed in Patients With Advanced Non-small Cell Lung Cancer or Patients With Head and Neck Cancer Who Are Treated With BI 754091 | No results posted | 21 (7.4%) pts had AEs leading to study drug discontinuation, most commonly IRRs in 6 (2.1%) pts. Serious AEs (all-cause) occurred in 77 pts (27.0%), most commonly pleural effusion in 6 (2.1%) and deep vein thrombosis in 4 (1.4%) pts. 2 pts (0.7%) experienced an AE resulting in death. |
| NCT03156114 | This Study Tests the New Medicine BI 754111 Alone or in Combination With Another New Substance BI 754091 in Patients With Advanced Cancer. The Study Tests Different Doses to Find the Best Dose for Continuous Treatment |  |  |
| NCT03433898 | This Study Aims to Find a Safe and Effective Dose of BI 754091. The Study Also Aims to Find Safe and Effective Doses of BI 754091 and BI 754111 in Combination. This Study is Done in Asian Patients With Different Types of Cancer |  |  |
| NCT03697304 | Platform Trial Evaluating Safety and Efficacy of BI 754091 Anti- PD-1 Based Combination Therapies in PD-(L)1 naïve and PD- (L)1 Pretreated Patient Populations With Advanced/Metastatic Solid Tumours |  |  |
| NCT02921685 | Study of a Humanized Antibody Initiated 2 Months After an HLA Matched Allogenic Stem Cell Transplantation (PIRAT) | No results posted | No results posted |
| NCT02671435 | A Study of Durvalumab (MEDI4736) and Monalizumab in Solid Tumors | In 17 pts receiving durvalumab plus monalizumab plus chemotherapy plus bevacizumab (DMCB), objective response rate was 41.2% (all PRs). | Most commonly fatigue, nausea, and peripheral neuropathy occurred in 100.0% of the DMCB and most commonly peripheral neuropathy, rash, and dermatitis acneiform in 94.1% of the durvalumab plus monalizumab plus chemotherapy plus cetuximab (DMCC). The grade 3/4 AEs in 77.8% of DMCB and 70.6% of DMCC and severe AEs in 38.9% DMCB and 47.1% DMCC. |
| NCT02643550 | Study of Monalizumab and Cetuximab in Patients With Recurrent or Metastatic Squamous Cell Carcinoma of the Head and Neck | Eight pts have a confirmed PR; ORR is 20% [95% confidence interval: 11-35]. Median time to response is 1.6 months. | In a phase I study, the combination of monalizumab and cetuximab was well tolerated. |
| NCT02459301 | A Dose-Ranging Study of IPH2201 in Patients With Gynecologic Malignancies | No results posted | No results posted |
| NCT02817633 | A Study of TSR-022 in Participants With Advanced Solid Tumors (AMBER) | No results posted | No results posted |
| NCT03680508 | TSR-022 (Anti-TIM-3 Antibody) and TSR-042 (Anti-PD-1 Antibody) in Patients With Liver Cancer | No results posted | No results posted |
| NCT03489343 | Sym023 (Anti-TIM-3) in Patients With Advanced Solid Tumor Malignancies or Lymphomas | No results posted | No results posted |
| NCT03311412 | Sym021 Monotherapy and in Combination With Sym022 or Sym023 in Patients With Advanced Solid Tumor Malignancies or Lymphomas | No results posted | No results posted |
| NCT03871855 | A Study of SHR-1702 Alone or With Camrelizumab in Participants With Advanced Relapsed/Refractory Solid Tumors | No results posted | No results posted |
| NCT03708328 | A Dose Escalation and Expansion Study of RO7121661, a PD-1/TIM-3 Bispecific Antibody, in Participants With Advanced and/or Metastatic Solid Tumors | No results posted | No results posted |
| NCT03066648 | Study of PDR001 and/or MBG453 in Combination With Decitabine in Patients With AML or High Risk MDS | No results posted | No results posted |
| NCT02608268 | Phase I-Ib/II Study of MBG453 as Single Agent and in Combination With PDR001 in Patients With Advanced Malignancies | No results posted | No results posted |
| NCT03099109 | A Study of LY3321367 Alone or With LY3300054 in Participants With Advanced Relapsed/Refractory Solid Tumors | Full target engagement (TE) was maintained 2 wks after 1200 mg dose; 600 mg Q2W maintained TE at steady-state. | No dose limiting toxicities, dose limiting-equivalent toxicities, treatment-related SAEs, or deaths were observed in LY3321367 monotherapy or combination therapy. |
| NCT03652077 | A Safety and Tolerability Study of INCAGN02390 in Select Advanced Malignancies | No results posted | No results posted |
| NCT03446040 | An Investigational Immunotherapy Study of BMS-986258 Alone and in Combination With Nivolumab in Participants With Solid Cancers That Are Advanced or Have Spread | No results posted | No results posted |
| NCT03744468 | Study of BGB-A425 in Combination With Tislelizumab in Advanced Solid Tumors | No results posted | No results posted |
| NCT03692429 | alloSHRINK - Standard cHemotherapy Regimen and Immunotherapy With Allogeneic NKG2D-based CYAD-101 Chimeric Antigen Receptor T-cells (alloSHRINK) | An encouraging clinical activity (2 pts with partial response and 9 with stable disease) | No results posted |
| NCT03466320 | DEPLETHINK - LymphoDEPLEtion and THerapeutic Immunotherapy With NKR-2 (DEPLETHINK) | No results posted | No results posted |
| NCT03370198 | Hepatic Transarterial Administrations of NKR-2 in Patients With Unresectable Liver Metastases From Colorectal Cancer (LINK) | No results posted | No results posted |
| NCT03310008 | Dose Escalation and Dose Expansion Phase I Study to Assess the Safety and Clinical Activity of Multiple Doses of NKR-2 Administered Concurrently With FOLFOX in Colorectal Cancer With Potentially Resectable Liver Metastases (SHRINK) | No results posted | No results posted |
| NCT03018405 | A Dose Escalation Phase I Study to Assess the Safety and Clinical Activity of Multiple Cancer Indications (THINK) | No results posted | No results posted |

Note: pts: patients; ORR: overall response rate; DCR: disease control rate; AEs: adverse events; iPR: partial response; DLTs: dose-limiting toxicities; cPRs: confirmed partial responses; SD: stable disease; IRRs: infusion-related reactions. Most data come from ASCO meeting library.
